# Phenotypic and Genotypic Traits of Pasteurella multocida subsp. septica Isolates From the Wounds of Two Patients Due to Dog or Cat Biting, 2023

**DOI:** 10.7759/cureus.42640

**Published:** 2023-07-29

**Authors:** Daisuke Taniyama, Takahiro Maeda, Takayuki Yokozawa, Akifumi Takano, Toshimi Oda, Sayori Li, Haruno Yoshida, Takashi Takahashi

**Affiliations:** 1 Department of Infectious Diseases, Showa General Hospital, Tokyo, JPN; 2 Laboratory of Infectious Diseases, Graduate School of Infection Control Sciences and Ōmura Satoshi Memorial Institute, Kitasato University, Tokyo, JPN; 3 Clinical Laboratory, Showa General Hospital, Tokyo, JPN; 4 Orthopedic Surgery, Showa General Hospital, Tokyo, JPN

**Keywords:** japan, 2023, dog/cat biting, traits, pasteurella multocida subsp. septica

## Abstract

We describe the phenotypic and genotypic traits of *Pasteurella multocida* subsp. *septica* isolates from the dog/cat bite wounds of two patients in 2023. A 79-year-old man with diabetes mellitus and cerebral infarction who was bitten by a dog on his left hand developed deep inflammation under the tendon between his left fourth and fifth fingers. The patient’s condition was resolved with antimicrobial treatment and surgical intervention. Another patient, a healthy 49-year-old woman who was bitten by a cat on her left hand, developed superficial inflammation of the left thumb and index finger. The patient’s condition improved with antimicrobial treatment without surgical intervention. The isolates from the two patients had similar biochemical properties, and the antimicrobial susceptibility data for both isolates indicated erythromycin resistance. Genotypic analyses revealed clade 2 on the dendrogram of repetitive sequence-based fingerprinting, capsule serogroup* cap *genotype A, and *hsf-1*-*nanH*-*pmHAS* (virulence-associated genes). Our observations show that the two isolates have similar phenotypic and genotypic traits, regardless of differences in patient background, biting pets, wound inflammation, or the necessity of surgical intervention.

## Introduction

*Pasteurella multocida* was first isolated by Pasteur in 1881 from an epidemiological case of fowl cholera. This species is a Gram-negative nonmotile coccobacillus that is found in many animals' oral cavities and gastrointestinal tracts (including those of cats and dogs). *P. multocida* is divided into three subspecies of multocida, septica, and gallicida, based on the internal sequences of the superoxide dismutase gene (sodA) with 16S rRNA gene sequences [1], because the 16S rRNA gene sequences are similar among *P. multocida*. In a bacteriological analysis of infected wounds caused by dog or cat bites, the Emergency Medicine Animal Bite Infection Study Group [2] found that *P. canis* (previously known as *P. multocida* biotype 6, revealing a positive ornithine decarboxylation test, a negative urease test, and variable acid production from trehalose and d-xylose) was the most common species isolated from dog bite wounds, whereas *P. multocida* subsp. multocida and *P. multocida* subsp. septica were the most common pathogens isolated from cat bite wounds. Herein, we report the phenotypic and genotypic traits of *P. multocida* subsp. septica isolates from the wounds of two patients bitten by a dog and a cat, respectively, in 2023.

## Case presentation

First case

**Figure 1:**
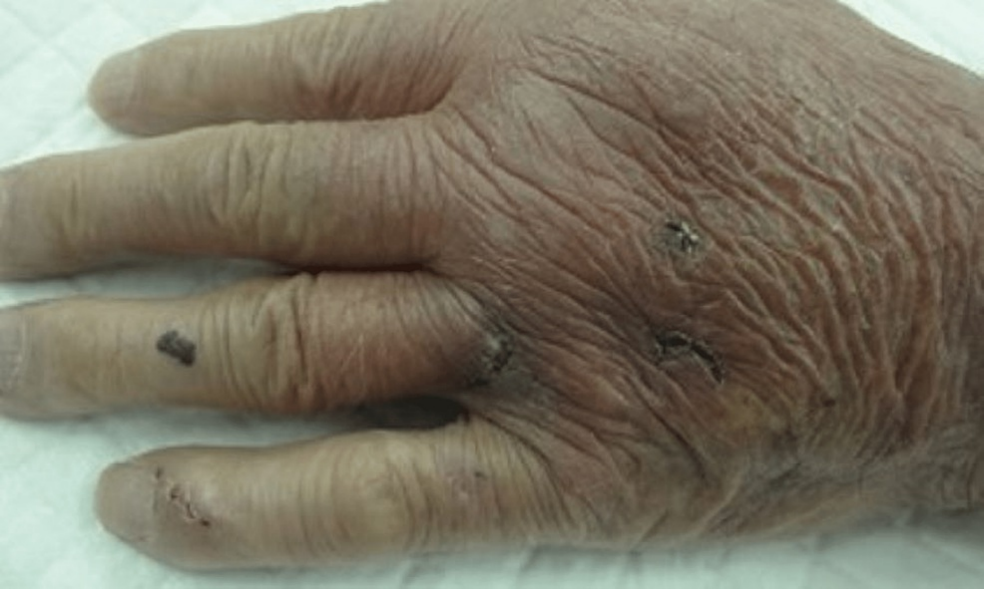
Appearance viewed from back of the hand of the first case

A 79-year-old man with diabetes mellitus and cerebral vascular infarction visited our emergency room in 2023. Two days before the visit, he had been bitten by a pet dog on his left hand. The fourth and fifth fingers of the left hand were swollen, and purulent discharge drained from the wounds. On admission, the patient had a temperature of 36.4 °C, a blood pressure of 132/76 mmHg, a respiratory rate of 14 breaths/min, an oxygen saturation of 97% (room air), and a heart rate of 118 beats/min. Physical examination was notable for swelling, redness, and tenderness in the fourth and fifth fingers (Figure 1).

Laboratory tests showed a white blood cell count of 11,700/mL, a urea nitrogen level of 21.1 mg/dL, a creatinine level of 1.09 mg/dL, a hemoglobin A1c level of 6.4%, and a glucose level of 155 mg/dL (Table 1).

**Table 1:** Investigation profile of the patient

Investigations	Case	Reference values
Hematology		
White blood cell	117×10^2^/μl	40.0-100.0×10^2^/μl
Red blood cell	491×10^4^/μl	420-550×10^4^/μl
Hemoglobin	14.6 g/dl	13.2–17.2 g/dl
Hematocrit	44.90%	39.4–49.8%
Platelet	23.5×10^4^/μl	15.0–40.0×10^4^/μl
Biochemistry		
Total protein	7.2 g/dl	6.3–8.2 g/dl
Albumin	4.0 g/dl	3.4–5.0 g/dl
Serum sodium	138 mEq/l	133–147 mEq/l
Serum potassium	4.1 mEq/l	3.5–4.7 mEq/l
Serum chloride	103 mEq/l	98–110 mEq/l
Blood urea nitrogen	21.1 mg/dl	8.0–21.0 mg/dl
Serum creatinie	1.09 mg/dl	0.61–1.04 mg/dl
Aspartate aminotransferase	15 IU/l	7–38 IU/l
Alanine aminotransferase	16 IU/l	8–40 IU/l
Alkaline phosphatase	57 IU/l	38–113 IU/l
C-reactive protein	12.52 mg/dl	0–0.5 mg/dl
Glucose	155 mg/dl	70–109 mg/dl
Hemoglobin A1c	6.1%	4.6–6.2%

The patient was diagnosed with purulent tendovaginitis. Two sets of blood cultures using the BacT/ALERT3D system (bioMérieux Japan Ltd., Tokyo, Japan) and aerobic/anaerobic pus cultures were obtained because the patient was immunocompromised. Gram stain of the exudate showed Gram-positive cocci and Gram-negative rods. Intravenous ampicillin/sulbactam (12 g/day) was initiated, and the patient underwent emergency surgery for an early-stage exploratory incision and drainage. The operative findings included non-purulent effusion and inflammation that seemed to undermine the tendon. A non-purulent effusion culture was obtained during surgery; however, no microorganisms were observed on Gram staining. As there was no obvious degeneration of the tendon, a tendon synovectomy was performed to remove the inflamed synovial membrane. The pus culture collected on admission grew *P. multocida*, *Cutibacterium acnes*, *Fusobacterium nucleatum*, and aerobic Gram-positive cocci, whereas no microorganisms grew on the two sets of blood cultures. Anaerobic *C. acnes* and *F. nucleatum* were identified using the Rapid ID32A test kit (bioMérieux Japan Ltd.). The effusion culture collected during surgery was positive for *P. multocida*. The patient’s condition improved, and he was discharged on day 11. Antimicrobial treatment was changed to a three-week course of oral amoxicillin/clavulanate (amoxicillin 1500 mg/day with clavulanate 375 mg/day). The patient remained well without recurrence or sequelae.

Second case

**Figure 2:**
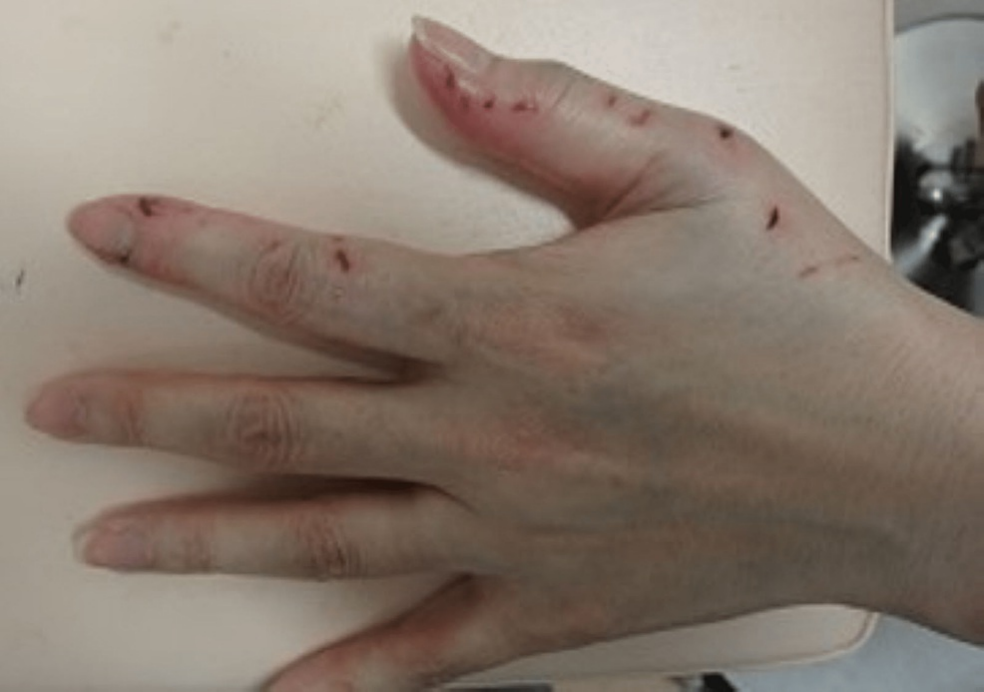
Appearance viewed from back of the hand of the second case

A healthy 49-year-old woman's left hand was bit by a pet cat seven hours before her presentation to our hospital in 2023. She presented with a swollen index finger and had a temperature of 36.1 °C, a blood pressure of 122/74 mmHg, a respiratory rate of 12 breaths per minute, an oxygen saturation of 98% (under room air), and a heart rate of 72 beats/min. Physical examination revealed swelling, redness, and tenderness of the left thumb and index finger (Figure 2).

Laboratory tests did not reveal any remarkable findings. A small volume of pus was draining from the index finger. A pus culture was obtained; however, no microorganisms were observed on the Gram staining. Cellulitis was diagnosed because the patient did not exhibit any Kanavel cardinal signs of flexor sheath infection. Oral administration of amoxicillin/clavulanate (amoxicillin 1500 mg/day with clavulanate 375 mg/day) was initiated as outpatient treatment. Careful observation of the patient confirmed that her condition had not worsened. The pus culture grew *P. multocida*, and the treatment was successful in resolving the redness. The patient remained well without recurrence or sequelae during the follow-up period of one month.

## Discussion

The phenotypic and genotypic traits are important when evaluating epidemiological evidence and associated virulence when selecting appropriate antimicrobial(s). Table 2 shows the phenotypic and genotypic traits of the *P. multocida *subsp.* septica* isolates (strains PA97 and PA98) from the wounds of our two patients who had been diagnosed with tendovaginitis (in the first case) and cellulitis (in the second case).

**Table 2:** Patient information and phenotypic/genotypic traits of Pasteurella multocida subsp. septica isolates from the wounds of two patients due to dog or cat biting in 2023 NCTC: National Collection of Type Cultures; soda: superoxide dismutase (manganese-dependent). Asterisk shows the different traits between PA97 and PA98 isolates. ID TEST HN-20 Rapid system (Nissui Pharmaceutical Co. Ltd., Tokyo, Japan) was used to assess the biochemical reactions except for sorbitol fermentation. API RAPID ID 32 E (bioMérieux Japan Ltd., Tokyo, Japan) was applied to evaluate the sorbitol fermentation. ^1^Key biochemical properties of *P. multocida subsp. septica* to distinguish from *P. multocida subsp. multocida* are shown. ^2^Antimicrobial susceptibility testing was done using disk diffusion and broth microdilution methods based on the Clinical and Laboratory Standards Institute document M45-A2 (2016). ^3^We included human-origin and animal-origin *P. multocida subsp. septica* (n = 18) with one human-origin *P. multocida subsp. multocida* and one human-origin *P. canis*. ^4^Virulence gene profile contained* hsf-1 *(autotransporter adhesion)*, pfhA *(filamentous hemagglutinin)*, toxA *(dermonecrotic toxin),* nanH *(small sialidase),* and pmHAS *(hyaluronidase).

Isolate no.	PA97	PA98
Patient age and se	79-year-old male	49-year-old female
Comorbidity	Diabetes mellitus, cerebral infarction	Healthy
Biting pet	Dog	Cat
Isolation year	2023	2023
Geographic location	Tokyo, Japan	Tokyo, Japan
Deep or superficial inflammation at the wound	Deep inflammation under the tendon between his left fourth and fifth fingers	Superficial inflammation of her left thumb and index finger
Surgical intervention	Needed	Not needed
Outcome	Cured	Cured
Similarity to 16S rRNA gene sequence of the NCTC 11995(T) (sequencing size)	99.72% (720 bp)	99.87% (757 bp)
Similarity to sodA gene sequence of the NCTC 11995(T) (sequencing size)	99.77% (429 bp)	99.77% (429 bp)
Urease activity	Negative	Negative
Ornithine decarboxylation	Positive	Positive
Indole production	Positive	Positive
Glucosidase activity	Positive	Positive
Mannitol fermentation	Positive	Positive
Trehalose fermentation^1^	Positive	Positive
Sorbitol fermentation^1^	Negative*	Positive*
Erythromycin susceptibility^2^	Resistant	Resistant
Fingerprinting by enterobacterial repetitive intergenic consensus sequences^3^	Clade 2	Clade 2
*Capsule serogroup cap* genotype	A	A
Virulence-associated gene profile^4^	hsf-1-nanH-pmHAS	hsf-1-nanH-pmHAS

The subspecies was identified based on both 16S rRNA gene and sodA sequencing data. Phenotypic analyses contained biochemical reactions (enzyme activities, various fermentations, ornithine decarboxylation, and indole production) and antimicrobial susceptibility testing (AST). The ID TEST HN-20 Rapid System (Nissui Pharmaceutical Co. Ltd., Tokyo, Japan) was used to assess the biochemical reactions [3], except for sorbitol fermentation, which was evaluated using the API RAPID ID 32 E (bioMérieux Japan Ltd.). AST was conducted using disk diffusion and broth microdilution methods based on the Clinical and Laboratory Standards Institute guidelines (document M45-A2). Genotypic analyses included fingerprinting by enterobacterial repetitive intergenic consensus sequences (ERIC)-based polymerase chain reaction (PCR) [4,5], *capsule serogroup cap* genotyping [6], and virulence-associated gene (VAG) profiling [7]. The VAG profile contained *hsf-1* (encoding autotransporter adhesion), *pfhA* (encoding filamentous hemagglutinin), *toxA* (encoding dermonecrotic toxin), *nanH* (encoding small sialidase), and *pmHAS* (encoding hyaluronidase). We included other human-origin and animal-origin *P. multocida* *subsp.* *septica* (n = 18) into the ERIC-PCR, with one human-origin *P. multocida* subsp. *multocida* (strain PA60) and one human-origin *P. canis* (PA57) as controls. Based on the PCR product images on 1.5% agarose gel, an unweighted pair group method with arithmetic mean (UPGMA) dendrogram was constructed using the Jaccard index for polymorphic DNA fingerprinting data. We used the DendroUPGMA (http://genomes.urv.cat/UPGMA/index.php) and NJplot (http://doua.prabi.fr/software/njplot) programs. Clustering clades were determined based on the dendrogram findings.

The biochemical properties of strains PA97 and PA98 were similar, except for sorbitol fermentation (Table 2). Table 3 depicts the AST data using the disk diffusion and broth microdilution methods against both isolates, along with erythromycin resistance, revealing similar AST patterns against PA97 and PA98.

**Table 3:** Antimicrobial susceptibility data against Pasteurella multocida subsp. septica isolates from the wounds of two patients due to dog or cat biting in 2023 Antimicrobial susceptibility testing was done using disk diffusion and broth microdilution methods based on the Clinical and Laboratory Standards Institute document M45-A2 (2016). Minimum inhibitory concentrations (μg/mL) of antimicrobials were determined using the broth microdilution method (Dry Plate Eiken DP44; Eiken Chemical Co., Ltd., Tokyo, Japan). There were similar antimicrobial susceptibility data against PA97 and PA98 isolates.

Isolate no.	PA97		PA98	
Antimicrobial susceptibility testing	Disk diffusion (susceptibility)	Broth microdilution (susceptibility)	Disk diffusion (susceptibility)	Broth microdilution (susceptibility)
Penicillin G		≤0.12		≤0.12
Ampicillin		0.5 (susceptible)		0.25 (susceptible)
Ampicillin/sulbactam		0.25/0.12		0.25/0.12
Amoxicillin/clavulanic acid	27 mm (susceptible)	≤0.25/0.12 (susceptible)	29 mm (susceptible)	≤0.25/0.12 (susceptible)
Cefazolin		2		1
Cefotaxime		0.25		0.12
Ceftriaxone	37 mm (susceptible)	≤0.25 (susceptible)	38 mm (susceptible)	≤0.25 (susceptible)
Cefepime		1		0.5
Cefdinir		≤0.25		≤0.25
Imipenem		0.5		0.25
Meropenem		≤0.06		≤0.06
Minocycline		≤1		≤1
Clarithromycin		4		4
Erythromycin	19 mm (resistant)	>2 (resistant)	22 mm (resistant)	>2 (resistant)
Azithromycin		1 (susceptible)		0.5 (susceptible)
Clindamycin		>2		>2
Moxifloxacin		≤0.5		≤0.5
Levofloxacin	31 mm (susceptible)	≤1	35 mm (susceptible)	≤1
Sulfamethoxazole/trimethoprim	28 mm (susceptible)	≤9.5/0.5 (susceptible)	25 mm (susceptible)	≤9.5/0.5 (susceptible)
Vancomycin		>2		>2

**Figure 3:**
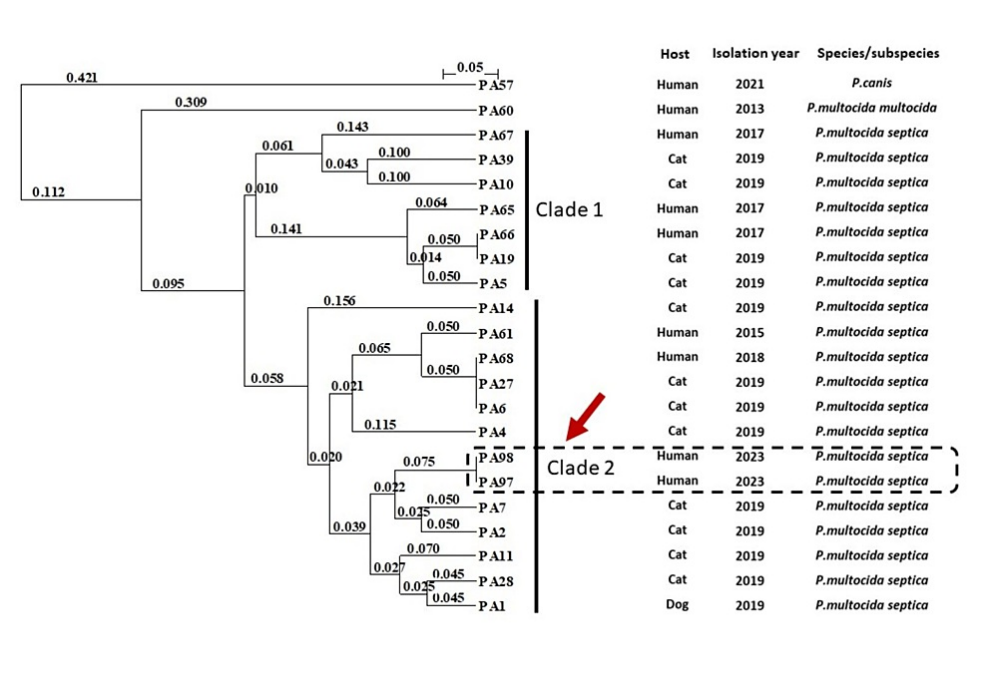
An unweighted pair group method with arithmetic mean (UPGMA) dendrogram of repetitive sequences-based fingerprinting When conducing enterobacterial repetitive intergenic consensus sequences (ERIC)-based polymerase chain reaction (PCR), we contained other human-origin and animal-origin *P. multocida* *subsp.* *septica* (n = 18) with one human-origin *P. multocida* *subsp.* *multocida* (strain PA60) and one human-origin *P. canis* (PA57) as controls. Based on the PCR product images on 1.5% agarose gel, an unweighted pair group method with arithmetic mean dendrogram was constructed using the Jaccard index for polymorphic DNA fingerprinting date by ERIC-PCR. We used the DendroUPGMA (http://genomes.urv.cat/UPGMA/index.php) and NJplot (http://doua.prabi.fr/software/njplot) programs. Clustering clades were determined based on the dendrogram findings. Dotted box shows PA97 and PA98. Different hosts and isolation years are indicated.

Additionally, genotypic analyses yielded the same cluster (clade 2) on the UPGMA dendrogram of ERIC-PCR fingerprinting (Figure 3) and indicated that cap genotype A contributed to the capsule serogroup and that the VAG profile contained *hsf-1-nanH-pmHAS*. PA60 and PA57 were found to be outliers on the UPGMA dendrogram.

An earlier study in Hungary is*olated 15 P. multocida* isolates from human patients (including 12 *P. multocida subsp. septica* isolates), characterized them using traditional and molecular methods, and compared them with feline isolates (n = 5) [8]. Nearly all isolates in this manuscript showed a similar VAG profile consisting of *hgbA*/*hgbB* (encoding iron acquisition proteins) and *nanH*. We should examine the presence/absence of *hgbA*/*hgbB* using two isolates as other VAG profiles. Additionally, these isolates were resistant to erythromycin and sulfamethoxazole but susceptible to ampicillin, whereas our isolates were resistant to erythromycin alone. The similar traits (VAG profile and AST pattern) found for isolates from humans and cats thus support the hypothesis that domestic cats are potential reservoirs for *P. multocida*. This indicates the need for the sequential determination of VAG profiles and AST patterns in human Japanese populations as well as their pet cats and dogs.

Hence, our observations show similar phenotypic and genotypic traits in two *P. multocida subsp. septica* isolates from the two isolates cultured from these two cases, despite the difference between the two cases in biting a pet (a dog or a cat). We searched the literature for related publications with the keywords "*P. multocida subsp. septica*, Japan" or "*P. multocida septica*, Japan" on the PubMed database (https://pubmed.ncbi.nlm.nih.gov/). However, our search yielded only one case report [9] (as of March 18, 2023). Further studies are needed to examine the similarities or differences in phenotypic and genotypic traits of clinical *P. multocida subsp. septica* isolates from a large number of cases.

## Conclusions

We specified the phenotypic and genotypic traits of *P. multocida* subsp. *septica* isolates from the wounds of two patients bitten by a dog and a cat in 2023. Physicians should perform cultures using the sterile specimens from infection foci (e.g., effusion collected during surgery in the first case) as well as the non-sterile specimens from infection foci (e.g., pus in the first case).
